# The Effectiveness of Eye Movement Desensitization and Reprocessing Toward Adults With Major Depressive Disorder: A Meta-Analysis of Randomized Controlled Trials

**DOI:** 10.3389/fpsyt.2021.700458

**Published:** 2021-08-06

**Authors:** Shuya Yan, Yanyan Shan, Shuming Zhong, Haofei Miao, Yange Luo, Hanglin Ran, Yanbin Jia

**Affiliations:** ^1^Department of Psychiatry, First Affiliated Hospital of Jinan University, Guangzhou, China; ^2^School of Management, Jinan University, Guangzhou, China; ^3^Psychology and Neuroscience Department, Duke University, Durham, NC, United States

**Keywords:** major depressive disorder, eye movement desensitization and reprocessing, meta-analysis, randomized controlled trial, cognitive behavioral theraphy

## Abstract

The practice-based evidence suggests that it is possible to use eye movement desensitization and reprocessing (EMDR) to treat major depressive disorder (MDD), but its specific efficacy is unknown. A systematic search was carried out for randomized controlled trials comparing EMDR with a control condition group in MDD patients. Two meta-analyses were conducted, with symptom reduction as primary outcome and remission as exploratory outcome. Eight studies with 320 participants were included in this meta-analysis. The first meta-analysis showed that EMDR outperformed “No Intervention” in decreasing depressive symptoms (standardized mean difference [SMD] = −0.81, 95% CI = −1.22 to −0.39, *p* < 0.001, low certainty), but statistically significant differences were not observed in improving remission (risk ratio = 1.20, 95% CI = 0.87–1.66, *p* = 0.25, very low certainty). The second showed the superiority of EMDR over CBT in reducing depressive symptoms (mean difference [MD] = −7.33, 95% CI = −8.26 to −6.39, *p* < 0.001, low certainty), and improving remission (risk ratio = 1.95, 95% CI = 1.24–3.06, *p* = 0.004, very low certainty). Besides, anxiety symptoms and level of functioning could not be included as secondary outcome due to the lack of data. The present meta-analysis suggests that EMDR is more effective in treating MDD than “No Intervention” and CBT, particularly in individuals who have traumatic experience. However, this result should be considered with caution due to small sample size and low quality of trails.

## Introduction

Major depressive disorder (MDD) is characterized by depressed mood, loss of interest, diminished ability to experience pleasure, and feelings of worthlessness or inappropriate guilt. According to the statistics released by the World Health Organization (WHO) in 2017, depression had affected more than 300 million people around the world, which means that 4.4% of the world's population is suffering from this disorder (1). In addition to emotional symptoms, MDD is also accompanied by a series of neurovegetative and cognitive symptoms (2). Given the severe impacts on quality of life and psychosocial functioning of affected people, MDD is deemed as the biggest contributor to global disability.

Psychological treatments have long been used to treat MDD. The latest clinical practice guideline of American Psychological Association (APA) recommends the use of cognitive-behavioral therapy (CBT) or interpersonal psychotherapy for the initial treatment of depression in adolescents. There was not enough evidence to recommend one psychotherapy treatment over another for adults and older adults with MDD, but in general, there was support for behavioral therapy; CBT and mindfulness-based cognitive therapy; and interpersonal psychotherapy. Among them, CBT is the most established evidence-based therapy. Multiple studies have found that CBT is effective in reducing depressive symptoms and preventing relapse when compared to usual care or placebo (3–5). For those with mild to moderate depressive symptoms, psychological intervention alone is proven effective (2). However, some patients still cannot fully benefit from them: after a full-session psychological therapy, only 53.7% participants can be evaluated as remission (6). There are various reasons why patients with MDD do not fully respond to psychotherapy. One of the reasons may be that the aforementioned interventions do not aim at particular clinical characterization of MDD patients, like early and/or recent environmental exposures (7). The distressing life experiences of MDD patients may not be effectively dealt with during interventions mentioned above, leading to unsatisfactory treatment outcomes.

Eye movement desensitization and reprocessing (EMDR) was derived from an accidental discovery by the North American psychologist Francine Shapiro in 1989: she found that spontaneous saccadic eye movements could magically lead to the reduction of distress brought by her disturbing memories (8). Nowadays, saccadic eye movements have developed into a standardized psychotherapy, containing patient history, preparation, assessment, desensitization, installation, body scan, closure, and re-evaluation. According to the Adaptive Information Processing (AIP) model, traumatic experience that cannot be fully processed will be stored in individuals' memory network in a frozen state (9). Such dysfunctional stored memories will enhance the chance of suffering from mental disorders (10, 11). While conducting eye movements during negative memories recall, the reprocessing of negative experience is facilitated, which leads to the relief of suffering.

EMDR was first employed in the treatment of post-traumatic stress disorder (PTSD). Two studies in 1989 demonstrated that saccadic eye movements could reduce frequency of traumatic memories and PTSD symptoms (8, 12). Over the past 30 years, EMDR has been considered as the first-line treatment of PTSD. Recently, studies revealed that EMDR can also be utilized in treating mental disorders that closely associated with distressing life experiences (13, 14). It is well-known that childhood trauma and stressful life events commonly present in MDD patients. According to a survey, about 55% of patients with MDD reported at least one type of childhood trauma (15). Besides, stressful life events are defined as a vital risk factor in the development and maintenance of MDD (16, 17). The presence of distressing life experiences in people with MDD may even prolong the disease course (18). In light of the close relationship between MDD and adverse events, researchers have started to apply EMDR in treating MDD (19–26).

Although there were reviews on the effectiveness of EMDR for MDD and affective disorders (13, 27–29), these reviews did not implement strict inclusion criteria, and some of the included studies were non-controlled trials, which may undermine the persuasiveness of research results. Besides, some reviews included studies on both adults and adolescents with MDD. The heterogeneity of study subjects may lower the reliability of review outcome. Therefore, the purpose of this study is to conduct a meta-analysis based on exhausted inclusion criteria and further determine the effectiveness of EMDR in the treatment of adults with MDD based only on RCTs.

## Methods

### Reporting Standards

This meta-analysis followed the Preferred Reporting Items for Systematic Reviews and Meta-analyses (PRISMA) statement for meta-analyses of RCT. The protocol for this systematic review and meta-analysis was pre-registered in the PROSPERO (CRD42021213881).

### Search Strategy

A comprehensive literature search of all articles published from the beginning of the database up to November 2020 was conducted through the Web of Science, Proquest, PubMed, Cochrane, and CNKI databases. The search used medical subject headings (MeSHs) terms including: “Eye Movement Desensitization Reprocessing” AND [“Depression” OR “Depressive Disorder” OR “Depressive Disorder, Major”], or keyword searches using [“EMDR” OR “Eye Movement Desensitization and Processing”] AND [“Depression” OR “Major Depressive Disorder” OR “Depressive Disorder”] and set a filter for the RCT studies only. Moreover, searches of the reference lists of the literature review or meta-analysis were also conducted.

### Eligibility Criteria

The eligibility criteria for the current meta-analysis were studies that had an RCT design and discussed the effectiveness of EMDR on depressive disorder. The including criteria were the following: (a) study participants were 18 years of age or older; (b) formal diagnosis of MDD should be made according to the *Diagnostic and Statistical Manual of Mental Disorders* [*DSM*] (up through DSM-V), or the *International Classification of Diseases* [*ICD*] (up through *ICD-10*) or *Chinese Classification and Diagnostic Criteria of Mental Disorders* [*CCMD*]; (c) studies were included if they reported EMDR as the psychotherapy (manualized treatment or less standard application); (d) control intervention including Treatment as Usual (TAU), waiting-list control, and studies comparing EMDR plus a co-intervention with the co-intervention alone (e.g., EMDR + antidepressant medications (ADMs) vs. ADMs). We excluded letters, reviews, case reports, study protocols, or studies without a control group. Studies were not restricted based on language. Titles and abstracts were separately screened by two independent researchers and full papers of eligible study trials were retrieved. Disagreements about inclusion were settled by discussion with the third author.

### Data Extraction

The information extracted from the articles was participant characteristics (sample size, age, and gender), diagnosis characteristics (criteria to diagnose and diagnosis), intervention characteristics (experiment and control group interventions' type, the amount of sessions, duration of each session, frequency in a week, and total time of therapy), and outcomes (outcome indicators and assessment tools). The number of participants was extracted from each article using the post-treatment assessment completers. Our primary outcome was reduction in depressive symptoms, and exploratory outcome was remission from depression evaluated by any assessment. Two authors independently carried out data extraction and any disagreements would be resolved by discussion.

### Risk of Bias Evaluation and Quality Assessment

All included articles underwent a risk of bias (RoB) assessment using the Cochrane Collaboration Tool for Assessing Risk of Bias in Randomized Trials (30). We assessed six domains of Selection bias, Performance bias, Detection bias Attrition bias, Reporting bias, and Other bias. However, since it is not feasible to blind participants and therapists during psychological interventions, we removed the criterion “Performance bias” and added the estimation of treatment implementation instead (31). The estimation of treatment implementation included therapist allegiance, treatment fidelity, and therapist qualifications (32, 33). Therapist allegiance refers to therapists' beliefs and preference for one treatment over another (34). Treatment fidelity refers to the degree to which treatments are implemented as intended (35). Therapist qualifications refer to whether therapists received training before implementation of therapy. Besides, the criterion “Other bias” was utilized to assess potential conflicts of interest. The evaluation of each domain could be low risk of bias, some concerns, or high risk of bias. Only trials assessed at low risk of bias within all assessed domains were classified as trials at overall low risk of bias.

The certainty of the evidence was evaluated with the use of the Grading of Recommendations Assessment, Development and Evaluation (GRADE) approach. RoB and certainty of evidence assessment were carried out by two independent researchers. Any disagreement was settled by discussion with the third author when necessary. We also contacted study authors to obtain missing data for the assessment of risk of bias.

### Statistical Analysis

The meta-analysis summarized and pooled statistics using the RevMan 5.4 software (36). For continuous outcomes, we calculated mean differences (MDs) with 95% CIs. When different scales were used to measure the same outcome type, we calculated standardized mean differences (SMDs) by dividing the MD between an EMDR and a control group by the pooled SD at the end of treatment. For dichotomous outcomes, we calculated risk ratios with 95% CIs. Differences were considered statistically significant at the *p* < 0.05 level. For all the outcome, fixed-effect and random-effects meta-analyses were both conducted and the most conservative result would be reported. If the results of two analyses were similar, then the result with widest CI would be chosen as our main result (37). The χ^2^ test of heterogeneity was simultaneously employed to confirm a fixed effects model.

### Trial Sequential Analysis

To control for risks of false-positive results (type I errors) and false-negative results (type II errors) because of repetitive testing of accumulating data, we conducted a trial sequential analysis (TSA) on each outcome. This analysis also estimated the information size needed to detect or reject an anticipated minimal clinically relevant difference between experimental and control groups. The TSA would be conducted in Trial Sequential Analysis Viewer 0.9.5.10 software (38).

## Results

### Description of Studies

**Figure 1:**
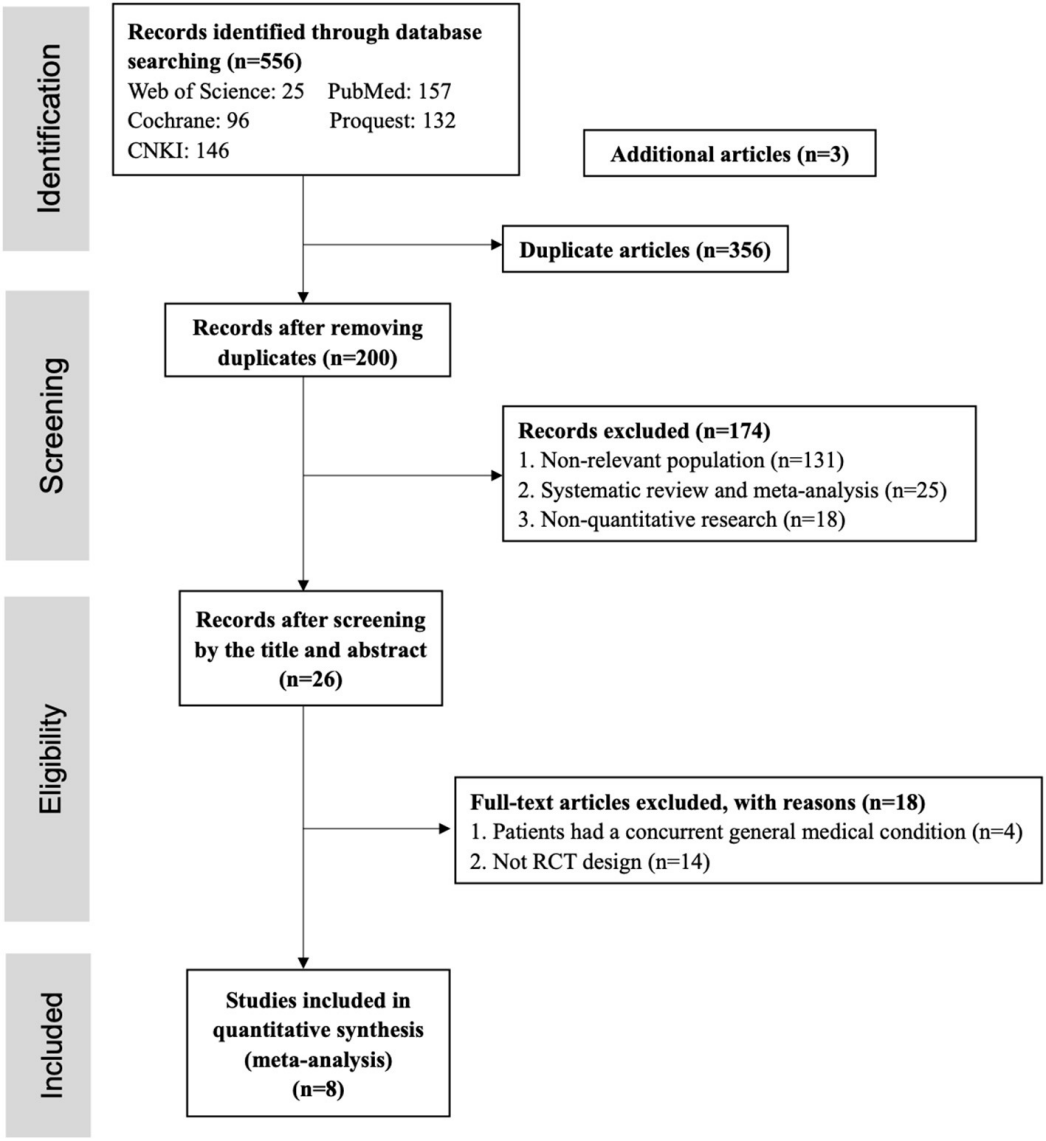
PRISMA flow diagram.

As the PRISMA flow chart shown in Figure 1, a total of 556 studies were screened during the systematic search and this number was reduced to 200 after removing duplications (*n* = 356). The remaining studies were screened by title and abstract; 174 studies were excluded with the following reasons: (1) the sample of studies was non-relevant population (*n* = 131); (2) systematic review and meta-analysis (*n* = 25); (3) non-quantitative research (*n* = 18). There were 26 studies screened by full text, and 18 studies didn't meet the inclusion criteria because: (1) in four studies, patients had a concurrent general medical condition; (2) 14 studies were not RCT design. Finally, eight studies were included and analyzed in this meta-analysis.

### Study Characteristics

Eight studies published from 2001 to 2020 were included in our meta-analysis, comprising 320 participants with depressive disorder (experiment group *n* = 159, control group *n* = 161). The sample was aged between 18 and 60 years old with mean age ranging from 29.38 to 52.85. Among these studies, four studies (20, 21, 25, 26) compared EMDR plus co-intervention with co-intervention (ADMs, CBT, and psychodynamic therapy, respectively). One study compared EMDR with waiting list (19). The remaining studies compared EMDR with CBT (including group CBT) (22–24). Based on the design of comparators, we divided trials into two comparisons: (1) EMDR vs. “No Intervention” (including waitlist and co-intervention, with a co-intervention delivered in both experimental and control groups); (2) EMDR vs. CBT. In almost all of the studies had a high percentage of female participants (Table 1).

**Table 1:** Characteristic of randomized controlled trials (RCTs) included in the meta-analysis (*N* = 8).

**No**	**Study citation**	**Participant diagnosis**	**Participants**	**Group type (E/C)**	**Intervention characterization** **Format/frequency**	**Outcome indicator & Measurement tools**	**Country**
1	Hogan (22)	**Criteria for diagnosis**	**Sample size** (total; E/C)	E:EMDR	**Format:** individual	**Depression:** BDI-II	UAS
		*DSM-IV*	**Total:** 30 (E:15; C:15)	C:CBT	**Frequency:**	**Symptoms:** SCL-90-R	
			**Gender** M:8; F:22		Session:1		
			**Mean age:** 39.7 (adult)		Duration: NM		
					Days of week: 1 × /week		
					Total week:1		
					Total time: NM		
2	Song and Wang (25)	**Criteria for diagnosis**	**Sample size** (total; E/C)	E:EMDR+ADM	**Format:** individual	**Depression:** HRSD	China
		CCMD-3	**Total:** 64 (E:32; C:32)	C: ADM	**Frequency:**		
			**Gender** M:29; F:35		Session:10		
			**Mean age:** 34.0 (adult)		Duration: 50 min		
					Days of week: 1–3 × /week		
					Total week:6		
					Total time: 20.8 h		
3	Gauhar (19)	**Criteria for diagnosis**	**Sample size** (total; E/C)	E:EMDR	**Format:** individual	**Depression:** BDI-II	Pakistan
		*DSM-IV*	**Total:** 26 (E:13; C:13)	C: waitlist	**Frequency:**	**Quality of life:** QLI	
			**Completed:** (E:10;C:7)		Session:6-8	**Traumatic Symptoms:** TSC-40	
			**Gender** M:7; F:10		Duration:1h		
			**Mean age:** 29.38		Days of week: 1 × /week		
			**Completed mean:** 29.4		Total week:7		
					Total time: 7 h		
4	Yu et al. (26)	**Criteria for diagnosis**	**Sample size** (total; E/C)	E:EMDR+ADM	**Format:** individual	**Depression:** HRSD	China
		*ICD-10*	**Total:** 90 (E:30; C:30) **Gender** M:43; F:47 **Mean age:** 32.07	C: ADM	**Frequency:** Session: 12 Duration: 50 min Days of week: 2 × /week Total week:6 Total time:10 h	**Life Events:** LES	
5	Ostacoli et al. (24)	**Criteria for diagnosis**	**Sample size**(total; E/C)	E:EMDR+ADM	**Format:** individual	**Depression:** BDI-II	Italy
		*ICD-10*	**Total:** 66 (E:31; C:35) **Gender** M:10; F:56 **Mean age:** 47.86	C: CBT+ADM	**Frequency:** Session:12–18 Duration: NM Days of week: 1 × /week Total week:3–6 months Total time: NM	**Global Functioning:** GAF **Quality of Life:** WHOQOL-Brief **Anxiety:** BAI	
6	Hase et al. (21)	**Criteria for diagnosis**	**Sample size** (total; E/C)	E: EMDR + psychodynamic/Behavioral group therapy	**Format:** individual	**Depression:** BDI-II	Germany
		*ICD-10*	**Total:** 30 (E:14; C:16)	C: psychodynamic/behavioral group therapy	**Frequency:**	**Symptoms:** SCL-90-R	
			**Gender** M:27; F:3		Session: 4–12		
			**Mean age:** 39.74		Duration: NM		
					Days of week: 1–2 × /week		
					Total week: 3–6 months		
					Total time: NM		
7	Minelli et al. (23)	**Criteria for diagnosis**	**Sample size** (total; E/C)	E:EMDR	**Format:** individual	**Depression:** BDI-II, MADRS	Italy
		*DSM-IV*	**Total:** 22 (E:12; C:10)	C: TF-CBT	**Frequency:**	**Anxiety:** BAI	
			**Gender** M:6; F:16		Session: 24	**Sleep Quality:** PSQI	
			**Mean age:** 52.85		Duration: 60 min		
					Days of week: 3 × /week		
					Total week: 8 weeks		
					Total time: 24 h		
8	Dominguez et al. (20)	**Criteria for diagnosis**	**Sample size** (total; E/C)	E: group CBT+EMDR	**Format:** individual	**Depression**:DASS-42	Australia
		DSM-5	**Total:** 45 (E:16; C:16)	C: group CBT	**Frequency:**	**Anxiety:** DASS-42	
			Completed: (E:15; C:16)		Session: 3		
			**Gender** M:20; F:29		Duration: 90 min		
			**Mean age:** 40.6 (adult)		Days of week: 1 × /week		
					Total week: 3 week		
					Total time:4.5 h		

*Diagnostics: DSM-IV, Diagnostic and Statistical Manual of Mental Disorders IV; DSM-5, Diagnostic and Statistical Manual of Mental Disorders 5; ICD-10, International Classification of Diseases 10*.

*Interventions: EMDR, eye movement desensitization and reprocessing; CBT, cognitive behavioral therapy; TF-CBT, trauma-focused cognitive behavioral therapy; ADM, antidepressant medication*.

*Instruments: BDI-II, Beck Depression Inventory-II; QLI, Quality of Life Index; TSC-40, Trauma Symptoms Checklist-40; HAMD, Hamilton Depression Scale; LES, Life Event Scale; GAF, Global Assessment of Functioning Scale; WHOQOL-Brief, WHO-Quality of Life Brief; BAI, Beck Anxiety Inventory; PSQI, Pittsburgh Sleep Quality Index; DASS-42, Depression, Anxiety and Stress Scale-42*.

### Risk of Bias

**Figure 2:**
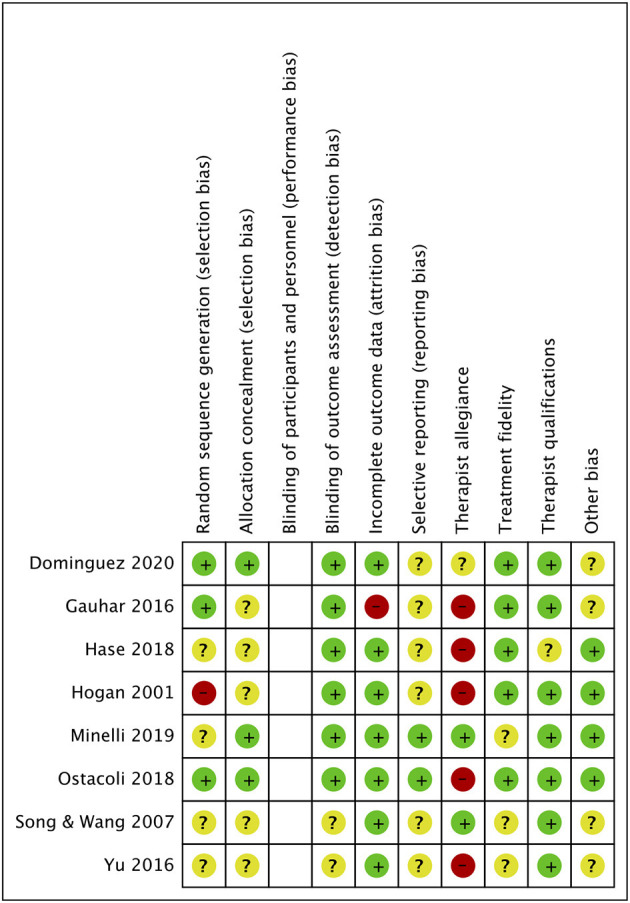
Risk of bias summary.

**Figure 3:**
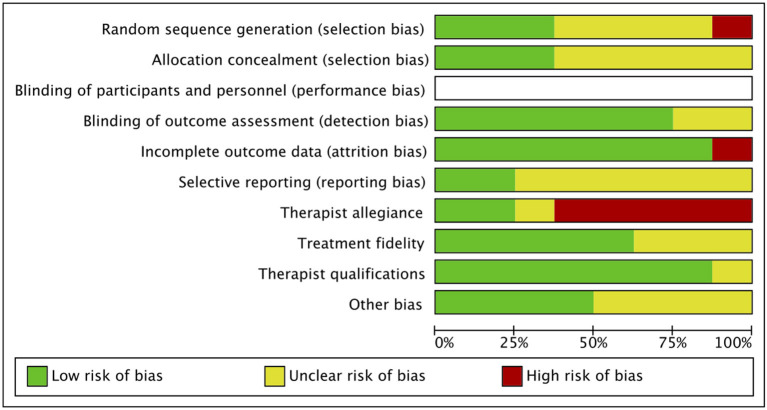
Risk of bias graph.

Figures 2, 3 showed the evaluation of risk of bias of studies included in this meta-analysis. In the criterion “Random sequence generation,” four studies (21, 23, 25, 26) that did not provide sufficient information of sequence generation were estimated as “some concerns.” One study (22) was rated as high risk because the generation of sequence may bring risk of bias to the outcome. Five studies (19, 21, 22, 25, 26) were rated as “some concerns” due to insufficient description of the method of concealment in the criterion “Allocation concealment.” Two trails (25, 26) did not report any information about the blindness of outcome assessors. One study (19) did not use effective analysis methods to correct the bias brought by missing data. Due to inadequate information about the expected outcomes and published reports, all the included studies were rated as “some concerns” in the criterion “Selective reporting,” except two studies (23, 24). Five studies (19, 21, 22, 24, 26) had a high risk of therapist allegiance, and one study (20) was rated as “some concerns.” Three studies (23, 25, 26) did not provide any information about treatment fidelity and one study (21) did not report the qualifications of therapists. As for conflict of interests, four studies were rated as “some concerns”: three studies did not provide any information (19, 25, 26) and one study (20) reported insufficient description of the role of funding in the study. All of the studies were rated as high risk of bias.

### Statistical Analyses

#### Comparison 1: EMDR vs. “No Intervention”

##### Primary Outcome: Reduction in Depressive Symptoms Severity

**Figure 4:**
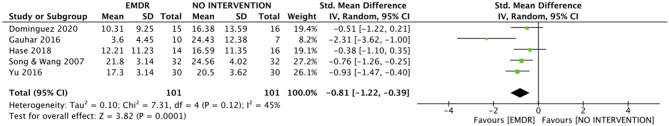
Forest plots of meta-analyses EMDR vs. “No Intervention” control on severity of MDD. EMDR, eye movement desensitization, and reprocessing. MDD, major depressive disorder.

Five studies with 202 participants (EMDR group *n* = 101, “No Intervention” group *n* = 101) examined the efficacy of EMDR in reducing depressive symptoms. One study used waiting list control (19), while four studies used co-intervention with CBT, ADMs, or psychodynamic therapy applied equally in both comparison groups (20, 21, 25, 26). Random-effects meta-analysis showed evidence of a difference in favor of EMDR vs. “No Intervention” group (SMD= −0.81, 95% CI = −1.22 to −0.39, *p* < 0.001) (Figure 4). TSA suggested that this result is unlikely to be a random finding due to lack of power or of multiple testing.

The *I*^2^ was 45% (χ^2^ = 7.31, *p* = 0.12), indicating a moderate but non-significant heterogeneity.

##### Exploratory Outcome: Remission From Depressive Symptoms

**Figure 5:**
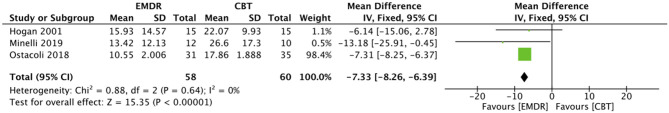
Forest plots of meta-analyses EMDR vs. “No Intervention” control on remission. EMDR, eye movement desensitization and reprocessing.

Three of five studies (*n* = 125) assessed the number of remissions from depressive symptoms at the end of EMDR in comparison with a co-intervention control group (CBT, ADMs, or psychodynamic therapy) (20, 21, 25). Among them, one study defined remission by scores reduction ≥75% of HAMD; one study used a BDI-II score <9 and the other study used a cut-off score of DASS to define remission. A total of 36 of 61 participants (59%) in the EMDR group, and a total of 31 of 64 participants (48%) in the “No Intervention” group were defined as remission. EMDR was more effective in improving remission than “No Intervention,” but statistically significant differences were not shown (RR = 1.20, 95% CI = 0.87–1.66, *p* = 0.25) (Figure 5). TSA could not be performed due to the small sample size.

The *I*^2^ was 0% (χ^2^ = 1.53, *p* = 0.46), indicating negligible heterogeneity.

#### Comparison 2: EMDR vs. CBT

##### Primary Outcome: Reduction in Depressive Symptoms Severity

**Figure 6:**
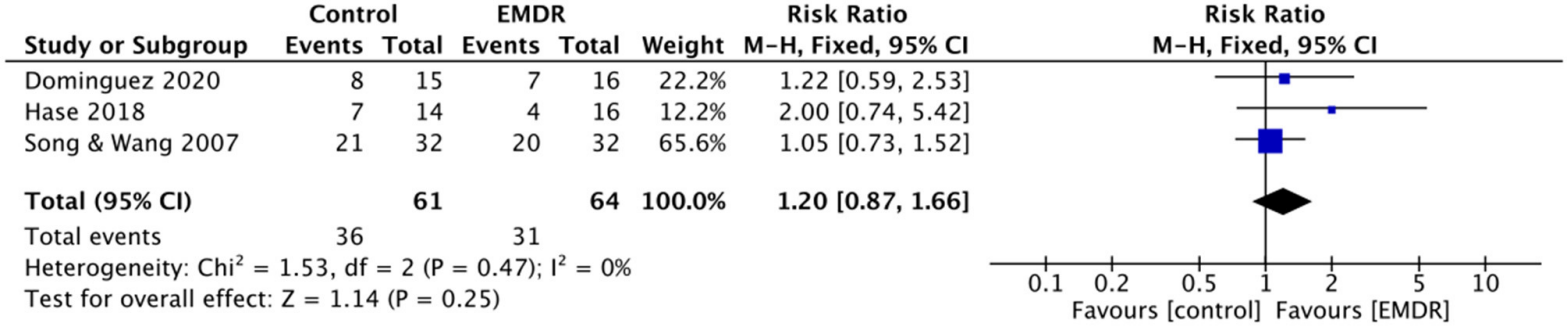
Forest plots of meta-analyses EMDR vs. CBT group on severity of MDD. EMDR, eye movement desensitization and reprocessing. CBT, cognitive behavioral therapy. MDD, major depressive disorder.

Three studies with 118 participants (EMDR group *n* = 58, CBT group *n* = 60) examined the efficacy of EMDR in reducing depressive symptoms on the BDI-II scale vs. CBT (22–24). Two studies applied conventional CBT (22, 24), while one study utilized trauma-focused CBT (TF-CBT) (23). Fixed-effect meta-analysis showed evidence of a difference in favor of EMDR (MD= −7.33, 95% CI = −8.26 to −6.39, *p* < 0.001) (Figure 6). TSA could not be performed due to the small sample size.

The *I*^2^ was 0% (χ^2^ = 0.88, *p* = 0.64), indicating negligible heterogeneity.

##### Exploratory Outcome: Remission From Depressive Symptoms

**Figure 7:**
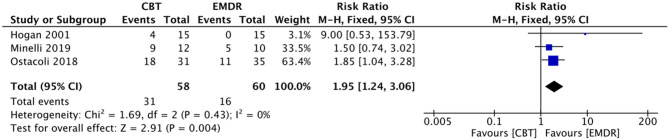
Forest plots of meta-analyses EMDR vs. CBT group on remission. EMDR, eye movement desensitization and reprocessing. CBT, cognitive behavioral therapy.

Three studies (*n* = 118) assessed the number of remissions from depressive symptoms at the end of EMDR in comparison with the CBT group. Among them, one study estimated remission from depressive symptoms according to the self-report of participants, one study used a BDI-II score <13, and the other study utilized an unstructured clinical interview to identify remission. A total of 31 of 58 participants (53%) in the EMDR group, and 16 of 60 participants (27%) in the CBT group were defined as remission. Fixed-effect meta-analysis showed that patients were less likely to have depressive symptoms in the EMDR group in comparison with the CBT group at the end of treatment (RR = 1.95, 95% CI = 1.24–3.06, *p* = 0.004) (Figure 7). TSA could not be performed due to the small sample size.

The *I*^2^ was 0% (χ^2^ = 1.69, *p* = 0.43), indicating negligible heterogeneity.

#### Other Subgroup Analysis

##### Primary Outcome: Reduction in Depressive Symptoms Severity

**Figure 8:**
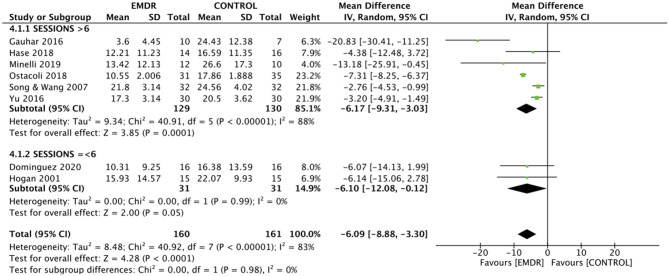
Effect of EMDR between sessions ≤ 6 and sessions >6 on severity of MDD. EMDR, eye movement desensitization and reprocessing; MDD, major depressive disorder.

The test for subgroup differences between sessions ≤ 6 and sessions >6 was not significant (χ^2^ = 0.00, *p* = 0.98). The MD in sessions >6 in favor of EMDR was −6.17 (95% CI = −9.31 to −3.03, *p* < 0.001); in session ≤ 6 was −6.10 (95% CI = −12.08 to −0.12, *p* = 0.05). Substantial heterogeneity was present in sessions >6 (*I*^2^ = 83%, *p* < 0.001), but not in sessions ≤ 6 (*I*^2^ = 0%, *p* = 0.99) (Figure 8).

#### GRADE Assessment

The GRADE assessments are presented in Table 2. The certainty of evidence was low or very low for all outcomes.

**Table 2:** Summary of findings tables for EMDR vs. “No Intervention” and EMDR vs. CBT.

**Outcomes**	Anticipated absolute effects* **(95% CI)**		**Relative effect (95% CI)**	**No of participants (studies)**	**Certainty of the evidence (GRADE)**	**Comments**
	**Risk with No Intervention**	**Risk with EMDR**				
**EMDR vs. “No Intervention” for MDD patients**						
Severity of MDD	-	SMD 0.81 SD lower (1.22 lower to 0.39 lower)	-	202 (5 RCTs)	⊕⊕○○ LOW^a^^,^^b^	EMDR may result in a large reduction in severity of MDD
Remission	484 per 1,000	581 per 1,000 (421–804)	RR 1.20 (0.87 to 1.66)	125 (3 RCTs)	⊕○○○ VERY LOW^a^^,^^b^^,^^c^	EMDR may not show preferable effect in reducing remission, but we are very uncertain
**Outcomes**	Anticipated absolute effects* (95% CI)		**Relative effect (95% CI)**	**No of participants (studies)**	**Certainty of the evidence (GRADE)**	**Comments**
	**Risk with CBT**	**Risk with EMDR**				
**EMDR vs. CBT for MDD patients**						
severity of MDD		MD 7.33 lower (8.26 lower to 6.39 lower)	-	118 (3 RCTs)	⊕⊕○○ LOW^a^^,^^b^	EMDR may show preferable efficacy in reducing severity of MDD compared to CBT
Remission	267 per 1,000	520 per 1,000 (331–816)	RR 1.95 (1.24 to 3.06)	118 (3 RCTs)	⊕○○○ VERY LOW^a^^,^^b^^,^^c^	EMDR may improve remission compared to CBT, but we are very uncertain

*The risk in the intervention group (and its 95% confidence interval) is based on the assumed risk in the comparison group and the relative effect of the intervention (and its 95% CI). CI, confidence interval; SMD, standardized mean difference; RR, risk ratio. The Grading of Recommendations Assessment, Development and Evaluation (GRADE) Working Group grades of evidence are as follows: High certainty, We are very confident that the true effect lies close to that of the estimate of the effect; Moderate certainty, We are moderately confident in the effect estimate: the true effect is likely to be close to the estimate of the effect, but there is a possibility that it is substantially different; Low certainty, Our confidence in the effect estimate is limited: the true effect may be substantially different from the estimate of the effect; Very low certainty, We have very little confidence in the effect estimate: the true effect is likely to be substantially different from the estimate of effect. ⊕○The symbol represents the grade of certainty. There are four grades of certainty: high certainty, moderate certainty, low certainty and very low certainty. The more number of “plus” symbol, the higher the grade of certainty. EMDR, eye movement desensitization reprocessing; SMD, standardized mean difference; RR, risk ratio; MD, mean difference; CBT, cognitive behavioral therapy*.

a *All trials were at an overall high risk of bias*.

b *The sample size was insufficient to calculate a precise effect estimate*.

c *Remission was assessed in non-standardized and potentially invalid manners*.

## Discussion

Eight randomized controlled trials were included in this meta-analysis of EMDR efficacy for adults with MDD. First, we conducted a comparison between EMDR and “No Intervention.” Our study revealed that EMDR was more effective in reducing depressive symptom than “No Intervention.” Second, we compared EMDR with CBT. The Primary and exploratory outcomes manifested that EMDR outperformed CBT in reducing depressive symptoms and enhancing remission. The subgroup analysis showed that there were no significant differences between sessions ≤ 6 and sessions >6.

The results of our study demonstrated that EMDR was superior to “No Intervention” in reducing depressive symptoms. Besides, three studies found that EMDR outperformed co-intervention control group in improving remission, but the differences between EMDR and the co-intervention control group failed to reach statistical significance. Currently EMDR is utilized to treat aversive memories and associated negative feeling and cognition. Of the included studies in this meta-analysis, about a half of MDD participants reported that they suffered from early or recent environmental exposures (19, 21, 23, 26). This may imply that EMDR can be used to treat this cohort.

The discussion about the mechanism of EMDR treatment has been around for a long time. The working-memory account is one of the accounts used to explain the role of eye movements in EMDR treatment. It posits that whenever patients recall past events, they will consume the processing resources of working memory at the same time. While performing eye movements during recall of negative memories, the processing resources are used up (39, 40). By occupying the expected processing resources of aversive memories, eye movements attenuate the vividness and emotionality of these negative memories. Because such memories appeared in a weakened form, patients will find that memories are not as horrible as they used to think, which means that the negative impact of past events also reduced accordingly (41).

Our findings suggested that EMDR was more effective than CBT in treating depressive symptoms and improving remission. To the best of our knowledge, this is the first meta-analysis to compare the efficacy of EMDR and CBT in the treatment of MDD in terms of symptom reduction and remission. The treatment target of EMDR is disturbing memories. Despite the fact that CBT also considers the impact of disturbing memories, it emphasizes the importance in shifting dysfunctional beliefs (42). Different treatment targets of these two psychological therapies may indicate that they are applicable in different clinical specific populations. In the trials that compared EMDR with CBT in our study, almost all of the participants reported stressful or traumatic experience, which implies that they may benefit more from trauma-focused psychotherapy like EMDR. Such psychotherapy can effectively deal with the influence of negative past events, which is a vital factor in the maintenance of current symptoms. Although EMDR and TF-CBT both belong to trauma-focused psychotherapy, one of the included trials reported an advantage for EMDR over another (23). The possible explanation may be the homework assignment, which is an essential component of CBT (43, 44). Compared with MDD patients without traumatic experience, those who underwent adverse events tended to report more severe symptoms (18), which may reduce their motivation to finish homework. The low compliance with homework completion may slow down the onset time of TF-CBT. In contrast, the efficacy and onset time of EMDR do not depend on homework assignment (9). Such characteristic may indicate that even if patients do not finish homework, the time required for EMDR to take effect will not be greatly affected. Hence, EMDR may work faster than TF-CBT for MDD patients in a given time, especially for those with severe symptoms.

We also conducted a subgroup analysis to investigate the effect of EMDR based on number of therapy sessions. However, we didn't find any significant difference. The finding of number of therapy sessions was consistent with the findings in patients with other mental disorders (14, 45, 46). The minimal number of therapy sessions in our study was one session (22), while the maximum number was 24, targeting treatment-resistant depression patients (23). Our result may imply that the efficacy of EMDR will not be restricted by therapy sessions because only the most distressing part of the incident, rather than the whole traumatic event, will be included as the treatment target during the treatment of EMDR (8, 9). The impact of the most distressing part is effectively dealt with in a short period of time. Hence, such targeted therapy makes it possible to relieve patients' symptoms even in a single session. Nevertheless, for those with severe symptoms, more treatment sessions are still necessary.

The aforementioned findings are consistent with the meta-analysis published earlier (27) and further confirm the effectiveness of EMDR on adult MDD patients. However, there are also several differences between the present meta-analysis and the one published before. The present meta-analysis included only RCT and applied strict inclusion criteria. RCT and strict inclusion criteria can provide plausible and strong evidence for the effectiveness of EMDR. Moreover, the present meta-analysis focused only on the adult cohort. The high homogeneity of study subjects makes our results more reliable and helps to promote the use of EDMR in adults with MDD. Last but not the least, besides studies conducted in western countries, the present study also included studies conducted in China, which indicates that the effectiveness of EMDR may not be influenced by the cultural background of patients.

There are several limitations in this meta-analysis. First, the number of included trials in this meta-analysis was small and the trials were rated as high risk of bias. It is recommended to conduct large well-designed RCTs to estimate the efficacy of EMDR in the future. Second, we did not have enough data to conduct subgroup analyses of anxiety symptoms and level of functioning. Only three trails reported the improvement of anxiety symptoms, and one trail provided information on level of functioning. Residual anxious and functional symptoms also play an important role in the recovery of adult MDD patients. Future studies are recommended to include anxiety symptoms and level of functioning as secondary outcomes in the investigation of the efficacy of EMDR in adult MDD patients. Third, the definition and measurement of remission in our studies were various, which may limit the comparability of the finding concerning remission assessed in other studies. A standardized semi-structure interview conducted by clinicians to identify remission is necessary in future studies.

## Conclusion

In general, notwithstanding the limitations of this meta-analysis, our study further confirmed the efficacy of EMDR in treating adults with MDD. We conducted two comparisons: (1) EMDR vs. “No Intervention” and (2) EMDR vs. CBT. Our findings suggested that EMDR was more effective in reducing depressive symptoms in comparison with “No Intervention” and CBT. Considering that most of the adult MDD patients had suffered from adverse experience, these findings may imply that EMDR has the potential to be an evidenced-based treatment for adults with depression, especially those with negative life events. However, these results should be considered cautiously due to the small sample size and methodological flaws. Further studies with high-quality design and large samples are needed to explore the efficacy of EMDR in treating adults with MDD and its long-term effects. Furthermore, including anxiety symptoms and level of functioning as secondary outcomes in the investigation of the efficacy of EMDR is also recommended in the future.

## Data Availability Statement

The original contributions presented in the study are included in the article/Supplementary Material, further inquiries can be directed to the corresponding author.

## Author Contributions

YJ designed the trial. SY, YS, and SZ conducted systematic research and acquired the data. SY and YS carried out the statistical analyses. SY drafted the initial manuscript. HM, YL, and HR interpreted the data. All authors revised the paper critically for important intellectual content, approved the final version, and agreed to be accountable for all aspects of the work. All authors contributed to the article and approved the submitted version.

## Conflict of Interest

The authors declare that the research was conducted in the absence of any commercial or financial relationships that could be construed as a potential conflict of interest.

## Publisher's Note

All claims expressed in this article are solely those of the authors and do not necessarily represent those of their affiliated organizations, or those of the publisher, the editors and the reviewers. Any product that may be evaluated in this article, or claim that may be made by its manufacturer, is not guaranteed or endorsed by the publisher.
