# Supplementary material for: The Effectiveness of Eye Movement Desensitization and Reprocessing Toward Adults With Major Depressive Disorder: A Meta-Analysis of Randomized Controlled Trials
Source: Front Psychiatry. 2021 Aug 6;12:700458. doi: 10.3389/fpsyt.2021.700458 (PMC8377362; doi:10.3389/fpsyt.2021.700458)
Supplement: Supplementary file 1 [file Data_Sheet_1.docx]

**Supplement 1** Trial Sequential Analysis and Diversity-Adjusted Required Information Size for The Effect of Eye Movement Desensitization and Reprocessing versus ‘No Intervention’ on Major Depressive Disorder Symptom Severity


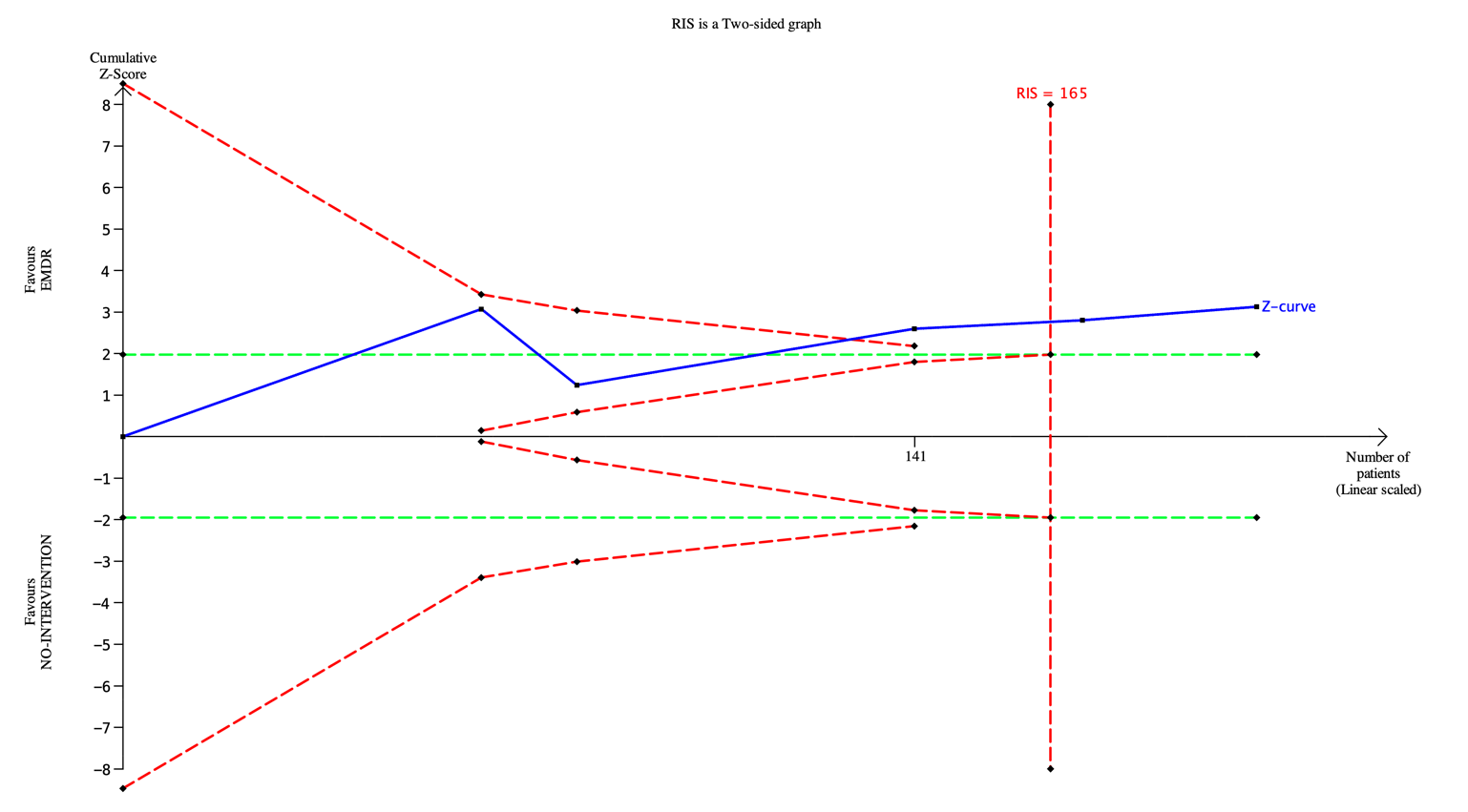


Note: The diversity-adjusted required information size for Beck Depression Inventory-II (BDI-II) and Hamilton Depression Scale (HAMD) was calculated to 165 patients based on a minimal relevant difference of 4.9 points, a standard deviation of 18.7, an alpha of 2.5%, a beta of 10%, and the observed diversity of 85%. Five trials with an accrued 202 patients reported results from BDI-II and HAMD. However, the cumulative Z-curve crossed the trial sequential monitoring boundary for benefit (outer wedge). The finding in the conventional meta-analysis of a statistically significant and substantial superiority of Eye Movement Desensitization and Reprocessing compared with ‘no intervention’ in reducing symptom severity is therefore unlikely to be a random finding due to lack of power or multiple testing if bias could be ignored. EMDR = Eye Movement Desensitization and Reprocessing
